# Research on path planning algorithms for Crawler transport robots in complex tunnels

**DOI:** 10.1371/journal.pone.0342122

**Published:** 2026-05-04

**Authors:** TongZhu Yu, JiuHong Wang, DaiXiang Zhang, YaoMeng Xiao, CanGuang Zheng, Dan Kang, Yang Jiang, MingChao Du, Kun Zhang, Xin Tong

**Affiliations:** 1 Yankuang Energy Group Co., Ltd, Jining, China; 2 Shandong University of Science and Technology, Qingdao, China; Qingdao University, CHINA

## Abstract

To meet the demand for efficient and safe underground material transportation in the intelligent construction of coal mines, addressing the unstructured environmental characteristics of mine roadways, this study proposes an improved path planning algorithm based on the A* algorithm. It aims to achieve the dual requirements of driving efficiency and operational safety for robots in complex environments. The algorithm adopts a collaborative architecture combining global and local path planning: at the global level, it enhances search efficiency by introducing a bidirectional adaptive search strategy and incorporates terrain risk weights into the cost function, enabling the planned path to effectively avoid high-risk areas and achieve global path optimization; at the local level, it integrates the DWA algorithm to strengthen the robot’s real-time obstacle avoidance capability and ensure operational safety. To validate the algorithm’s effectiveness, path planning experiments were conducted in a simulated environment. The results demonstrate that the proposed algorithm effectively avoids various obstacles, significantly shortens path length and search time, providing a viable solution for path planning and navigation of tracked transport robots in complex roadways.

## 1. Introduction

With the advancement of science and technology and the continuous progress of intelligent coal mine construction, the manual handling-dominated material transfer method for anchorage materials in the complex working environment of the excavation face clearly fails to meet the requirements of safe, efficient, and intelligent mining. Therefore, exploring the use of equipment-assisted or automated manual handling, and developing a transfer robot suitable for the complex working conditions of the excavation face can effectively improve underground workers’ operational modes, reduce labor intensity, enhance material loading efficiency, and become a key objective in intelligent coal mine construction [1]. However, traditional path planning algorithms face limitations in real-time dynamic obstacle avoidance, environmental adaptability, and global path optimization. Moreover, existing research primarily focuses on global path optimization in static environments, and the collaborative processing mechanism for kinematic constraints under dynamic disturbances and complex terrains remains imperfect. Therefore, achieving safe, efficient, and stable autonomous path planning in an environment where dynamic disturbances and terrain uncertainties coexist has become a crucial technical issue that needs to be addressed for the engineering application of tracked transfer robots.

In the field of automatic navigation, path planning, as the core technology is usually divided into global path planning and local path planning. Global path planning is to plan a global optimal or near optimal path from the starting point to the target point according to the given starting point and target point under the known global map information of the environment. In the global path planning algorithm, the traditional A* algorithm was proposed by Hart et al. [2] in the 1970s, and the heuristic A* algorithm proposed by Jeauneau V et al. [3] calculates the moving cost through the evaluation function and gradually extends to the target point. Wangzhiqiang et al. [4] improved the traditional A* algorithm from two aspects of search neighborhood and search direction, adaptively selected 4-neighborhood or 8-neighborhood search strategy, and developed an adaptive search direction A* algorithm on this basis. Gujianping et al. [5] designed an optimal path search model based on the optimized genetic algorithm. Nam [6] when calculating the cost function value of A* algorithm, the factor of turning angle cost is included. In this way, the planned path will more meet the actual driving demand of heavy transport vehicles with high turning cost. Akshay [7] changed the calculation of the heuristic value of A* algorithm from the initial stage to the collision stage, and the simulation shows that this method can significantly reduce the time required for planning the path. When Liu [8] designed the heuristic function of A* algorithm, he established the risk model of berths and maneuverability restrictions of ships to improve the navigation safety of the path. Sandip et al. [9] proposed the MHA* algorithm with multiple heuristic functions, which is suitable for the situation that a single heuristic function cannot describe the complexity of the problem. The algorithm calls all heuristic functions at the same time to find the optimal solution of the problem. Xie G et al. [10] proposed a new strategy combining Bessel curve and genetic algorithm, adding the limit of path and the risk value of path in the fitness function, and improving the selection operator and mutation operator. This new strategy can provide an effective path planning method to avoid risk points.

Local path planning, also known as real-time path planning or online path planning, is mainly used for path planning in the current local working environment of robots or vehicles in the process of movement. Ataollahi M [11] proposed a repulsion mechanism based on the force field model to avoid obstacles. Sheng z [12] and others combined and improved the BI-RRT* bidirectional exploration method and APF-RRT* expansion guidance, and proposed a bi-directional fast exploration random tree algorithm based on adaptive mechanism and artificial potential field (AB-APF-RRT*), which improved the RRT* sampling and expansion method. Das et al. [13] proposed a new algorithm for multiple robots based on the artificial potential field method. The algorithm can navigate from the source to the target position in a coordinated and formed way at the best path and time, so as to effectively avoid static and dynamic obstacles. Dynamic window approach (DWA) [14] as a commonly used local path planning algorithm, has good performance in dynamic obstacle avoidance. Zhang Rui et al. [15] proposed the RRT*-DWA collaborative algorithm, which uses the optimal random tree path as the tracking benchmark of the dynamic window method. Although it improves the dynamic environment adaptability, there is still the problem of obstacle avoidance response lag in high-density obstacle scenes. Therefore, LiuA et al. [16] proposed a trajectory similarity evaluation function based on the dynamic time warping method to provide better guidance; At the same time, aiming at the problem that DWA has poor adaptability to complex environment due to the fixed weight of evaluation function, a fuzzy controller is designed to enhance the adaptability of DWA algorithm in complex environment. Gong x et al. [17] improved the cost function of DWA algorithm to improve the efficiency of obstacle avoidance because of the problem that DWA algorithm cannot balance security and speed due to fixed parameters in complex environments.

In addition, path planning research has evolved from a focus on single algorithms to the integration of multiple strategies, and from static environmental assumptions to dynamic adaptability. In terms of dynamic adaptability, the research emphasis has shifted from planners with fixed parameters to models that can self-adjust based on environmental states. He Yong et al. [18] adjusted the weights of the objective function according to the distance and density of obstacles relative to the robot. Li Xinying et al. [19] employed the Multi-Objective Particle Swarm Optimization (MOPSO) algorithm to transform the adaptive change of DWA weight coefficients into a multi-objective optimization problem for solution. In terms of interference suppression and robustness enhancement, Zhang M et al. [20] introduced the Discrete-Time Control Barrier Function (DCBF) constraint to enhance the safety of obstacle avoidance for dynamic obstacles. At the algorithm framework level, there have emerged asymptotic optimal replanning algorithms specifically designed for dynamic environments, such as FMTX [21], which achieves efficient interference response by updating the incremental graph mechanism, repairing only the local path affected by environmental changes.

To sum up, considering the characteristics of narrow structure, dense arrangement of equipment, random distribution of temporary obstacles and dynamic changes in geological conditions of coal mine roadway props, the traditional path planning algorithm has obvious limitations in real-time obstacle avoidance ability, dynamic environment adaptability and global optimization, and the existing research mostly focuses on the global path optimization in the static environment, while the collaborative processing mechanism for the unique dynamic disturbance factors of the heading face, such as mobile equipment or personnel inrush, as well as the minimum turning radius and other constraints, has not been improved. To solve the above problems, this paper proposes a bilevel path planning framework combining the improved A* algorithm and the dynamic window method (DWA): firstly, the A* algorithm based on the improved cost function realizes the global optimal path search, secondly, the dynamic window method (DWA) is used for local dynamic obstacle avoidance, and the multi-objective constraint optimization model is established by combining the real-time environmental perception data of inertial navigation and lidar. To verify the engineering applicability of the algorithm, the simulation model of coal mine roadway was built in the gazebo simulation platform in ros1, and the navigation experiment was completed in the simulation environment [22].

## 2. Research on an improved path planning algorithm based on the A* algorithm

### 2.1. Bidirectional alternating search strategy

**Fig 1:**
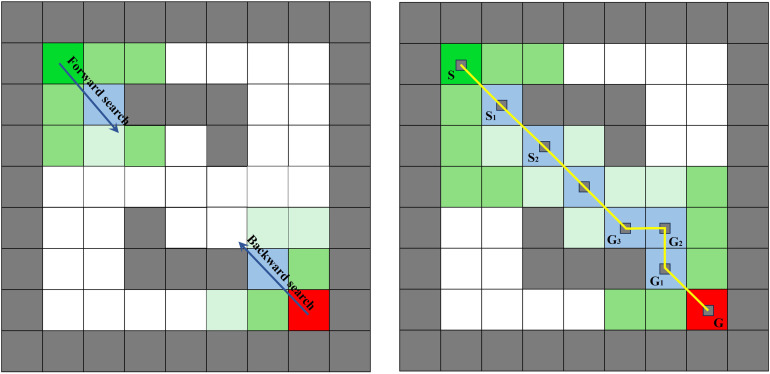
Schematic diagram of bidirectional search strategy planning.

Addressing the computational efficiency bottleneck issue caused by the unidirectional traversal strategy in the traditional A* algorithm, this paper introduces a bidirectional adaptive search strategy [23] for optimization. This approach establishes a bidirectional alternating expansion path exploration mechanism between the starting point and the end point, significantly improving the algorithm’s search efficiency and environmental adaptability while reducing the frequency of redundant node traversal. The specific process of this strategy is as follows: First, allocate and clear the forward search queue (F_OPEN) and the backward search queue (B_OPEN), and reset the node closure set (CLOSE) and the node traceability matrix (PARENT) [24], thereby establishing a basic framework for bidirectional collaborative search. Subsequently, select the node with the minimum evaluation function value from the forward and backward OPEN lists as the current expansion node, denoted as N_F_ and N_B_ respectively, and move them into their corresponding CLOSE lists. During the forward and backward search operations, in the forward search phase, the target is the backward current node N_B_, and the adjacent nodes of the forward current node N_F_ are expanded, calculating the cost value of each adjacent node. If the node is an obstacle or already in the closure list, it is ignored. The parent node is updated to N_F_, and the forward open list is refreshed. In the backward search phase, the target is the forward current node N_F_, and the same process is executed correspondingly, filtering and updating the adjacent nodes of N_B_. After each expansion, update the new node that meets the conditions as the current node in the corresponding direction. Check whether the forward current node N_F_ and the backward current node N_B_ coincide. If they coincide, it indicates successful convergence of the bidirectional search. Then, through the bidirectional PARENT list, trace back from the convergence point to the starting point and the end point respectively, concatenate to obtain a complete path and output it. If they do not coincide, continue the search. If both the forward and backward OPEN queues are not empty during the search, continue the subsequent search; if any queue is empty, it indicates that the bidirectional search has traversed all possible nodes but failed to converge, and the search is determined to have failed and the program is terminated. Based on the above process, it can effectively avoid the back-and-forth oscillation phenomenon that occurs in unidirectional search under environmental obstacles, significantly reducing the number of expansion nodes. Its specific performance is shown in Fig 1.

In this study, the Euclidean distance function was used as the heuristic function, and the forward heuristic function *h*_*F*_(*N*_*F*_) and the backward heuristic function *h*_*B(*_*N*_*B)*_ were defined, where $\left(x_{N_{F}},y_{N_{F}}\right)$ and $\left(x_{N_{B}},y_{N_{B}}\right)$ are the coordinate values of the forward current search node N_F_ and the backward current search node N_B_, respectively. The specific expressions are shown in Eqs. (1) and (2):


$$h_{F}\left(N_{F}\right)=\sqrt{(x_{N_{B}}-x_{N_{F}}{)}^{\text{2}}+(y_{N_{B}}-y_{N_{F}}{)}^{\text{2}}}$$ (1)

$$h_{B}\left(N_{B}\right)=\sqrt{(x_{N_{F}}-x_{N_{B}}{)}^{\text{2}}+(y_{N_{F}}-y_{N_{B}}{)}^{\text{2}}}$$ (2)

**Fig 2:**
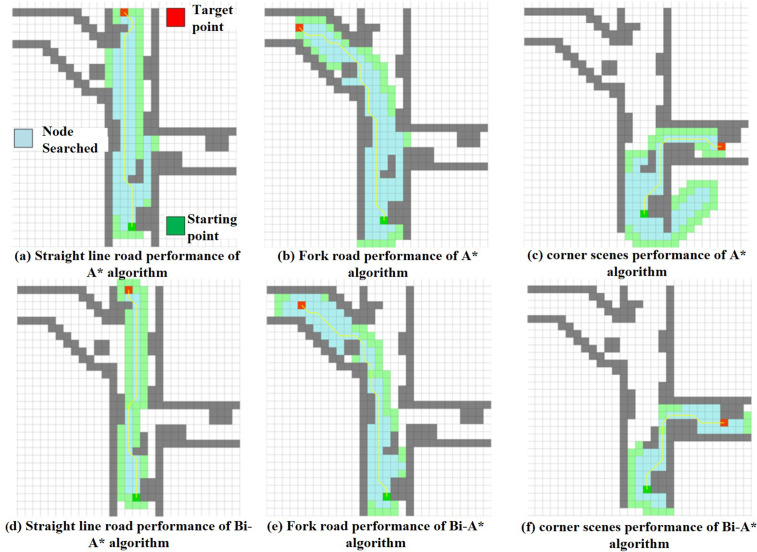
Comparison of Bi-A* algorithm and traditional A* algorithm in path planning.

To verify the efficiency of the bidirectional search strategy, simulation experiments were conducted based on MATLAB 2024b. These experiments tested the performance of the Bi-A* algorithm [25] and the traditional A* algorithm in generating collision-free paths under different workspaces, as shown in Fig 2. This figure includes six sub-figures. The upper row (a)(b)(c) depicts the search simulations of the traditional A* algorithm in three scenarios, while the lower row (d)(e)(f) illustrates the search simulations of the Bi-A* algorithm in corresponding scenarios. In the figure, dark green squares represent the “starting point”, red squares represent the “target point”, light blue squares indicate “searched nodes”, and gray squares represent obstacles in the scene. As can be seen from the figure, the upper half shows the simulation process of the traditional A* algorithm searching for the target point in scenarios involving straight lines, bifurcations, and corners. The lower half presents the search situation of the Bi-A* algorithm in the same scenarios. In the straight-line scenario, the traditional A* algorithm performs a unidirectional search from the starting point to the target point, with the light blue searched nodes covering a longer path and a wider search range. The Bi-A* algorithm starts searching from both the starting point and the target point simultaneously, resulting in a more concentrated coverage of searched nodes, a simpler search path, and a significant reduction in the number of nodes. In the bifurcation scenario, unidirectional search requires traversing more branch nodes, with the light blue area covering a large range and resulting in lower search efficiency. Bidirectional search can reduce the exploration of invalid branches, concentrating the searched nodes on effective paths, significantly reducing the number of search nodes, and improving efficiency. When facing corner terrain, unidirectional search requires gradual path exploration, with the light blue node coverage area being scattered, leading to a redundant search process. Bidirectional search converges faster at corners, concentrating the searched nodes on key paths, resulting in higher search efficiency, further verifying the optimization effect of the bidirectional search mechanism on path search in complex terrain.

The simulation results of traditional A* algorithm and Bi-A* algorithm are shown in Table 1. According to the table, the search time of Bi-A* algorithm is reduced by 10.5–33.3%, and the number of search nodes is reduced by 13.5–37.3%; At the same time, through the analysis of the experimental data, it can be seen that with the expansion of the environment scale, the calculation time of Bi-A* decreases more significantly than that of the traditional A* algorithm.

**Table 1:** Comparison of search data between Bi-A* algorithm and traditional A* algorithm.

Road condition	Algorithm	Time	Search node	Time saving relative to A*	Node saving relative to A*
a	A*	8.7	198	—	—
	Bi-A*	5.8	124	33.3%	37.3%
b	A*	8.5	244	—	—
	Bi-A*	7.6	211	10.6%	13.5%
c	A*	7.9	185	—	—
	Bi-A*	6.5	124	17.7%	33.0%

### 2.2. Improved cost function

Compared to two-dimensional path planning, when conducting actual path planning, the actual cost g(n) must not only consider the X, Y directions but also incorporate terrain risks to ensure that the planning results are more aligned with real-world application scenarios. Therefore, the design of weights is crucial, requiring a balance between reasonably penalizing terrain features and adhering to the motion characteristics of tracked robots.

The actual terrain slope and the basic movement cost (distance or energy consumption) exhibit significant differences in dimension and numerical range, necessitating normalization to eliminate the impact of dimension. Although tracked robots possess a certain degree of climbing capability, it significantly increases energy consumption. Let the slope angle between node n and its parent node be θ(n), with a range set between 0°and 30°. The formula for the slope angle is presented in Eq. (3):


$$\theta \left(n\right)=arctan\left[\frac{z\left(n\right)-z\left(n_{parent}\right)}{d\left(n_{parent},n\right)}\right]$$ (3)

In Eq. (3), *z*(*n*) and *z*(*n*_*parent*_) represent the height values of the current node *n* and its parent node *p*, respectively. *d*(*n*_*parent*_, *n*) denotes the horizontal movement distance (Euclidean distance) between the current node *n* and its parent node *p*. The calculation formula is presented in Eq. (4):


$$d\left(n_{parent},n\right)=\sqrt{{\left[x\left(n\right)-x\left(n_{parent}\right)\right]}^{\text{2}}+{\left[y\left(n\right)-y\left(n_{parent}\right)\right]}^{\text{2}}}$$ (4)

In Eq. (4), (*x*(*n*), *y*(*n*)) and (*x*(*n*_*parent*_), *y*(*n*_*parent*_)) represent the coordinate values of the current node *n* and its parent node *p*, respectively. To eliminate the issue of dimensional differences, a slope normalization function is designed. The actual slope angle *θ*(*n*) is divided by the maximum safe slope angle *θ*_*max*_(*n*) and normalized to a proportional coefficient within the interval [0, 1]. This coefficient can be directly multiplied by the basic cost (such as distance). Its nonlinear growth characteristic makes the algorithm more sensitive to steep slopes. The specific calculation formula is shown in Eq. (5):


$$f\left(\theta \right)=\left(\frac{\left|\theta \left(n\right)\right|}{\theta_{max}\left(n\right)}\right)$$ (5)

To quantify the energy consumption differences of tracked robots on different slopes, a segmented nonlinear penalty function is adopted to assess the slope risk. This function establishes a medium slope threshold *θ*_*middle*_: if the current slope angle *θ* exceeds *θ*_*middle*_, a high penalty coefficient *P*_*high*_ is applied, causing the penalty cost to increase rapidly; if the current slope angle *θ* is less than *θ*_*middle*_, a low penalty coefficient Plow is used; if *θ* exceeds the maximum safe slope angle, it is considered an impassable area. The specific calculation formula for the penalty function is shown in Eq. (6):


$$p\left(\theta \right)=\left\{ \begin{array}{cc} @cc@f\left(\theta \right)\cdot P_{low} & \theta <\theta_{midle}\\ f\left(\theta \right)\cdot P_{high} & \theta_{midle}\leq \theta \leq \theta_{max}\\ \infty & \theta >\theta_{max} \end{array}\right.$$ (6)

Considering computational efficiency, and to avoid introducing too many parameters that could affect path planning efficiency, the cost function design relies solely on the slope angle to simplify the algorithm and enhance its practicality. The improved cost evaluation function is presented in Eq. (7):


$$g_{i}\left(n\right)=g\left(n\right)\cdot \left[1+p\left(\theta \right)\right]$$ (7)

The cost function *g*_*i*_(*n*) incorporating slope weights, as defined by Eq. (7), is incorporated into the A* algorithm, forming an improved version of the A* algorithm. This algorithm effectively bypasses high terrain risk areas during the search process and seeks the globally optimal path, thereby enhancing overall search efficiency. The improved forward and backward search cost functions are presented in Eqs. (8) and (9), respectively:


$$f_{F}\left(n\right)=g_{i}\left(n\right)+h_{F}\left(N_{F}\right)$$ (8)

$$f_{B}\left(n\right)=g_{i}\left(n\right)+h_{B}\left(N_{B}\right)$$ (9)

### 2.3. DWA fusion strategy

Based on the traditional A* algorithm, the algorithm in this study introduces a piecewise slope penalty function into the cost function. By calculating the slope angle and performing normalization processing, it achieves the quantification and avoidance of terrain risks. The traditional DWA algorithm samples in the velocity space (*v*, *ω*) to predict multiple trajectories for a future period and selects the optimal trajectory based on the evaluation function. Therefore, to integrate the terrain risk into the DWA algorithm, the evaluation function of DWA is extended by adding a terrain slope cost term. The specific formula is shown in Eq. (10):


$$G\left(T\right)=\alpha \cdot Pdist\left(T\right)+\beta \cdot Gdist\left(T\right)+\gamma \cdot D_{obs}\left(T\right)+\delta \cdot terrain\left(T\right)$$ (10)

In the above formula, *Pdist* represents the degree of conformity between the local path and the global path, *Gdist* denotes the closeness between the path point and the target point, *D*_*obs*_ signifies the distance between the current target point and the obstacle, and *terrain* stands for the terrain slope cost value. *α*, *β*, *γ*, and *δ* are the weight coefficients for each cost, which can be determined by combining static presetting with dynamic adaptation according to the environmental state. In static or structured scenarios, fixed weights calibrated empirically can be adopted; whereas in complex environments such as mine tunnels, to adapt to sudden terrain changes and dynamic appearance of obstacles, the weights can be fine-tuned during the planning process based on real-time environmental information. For instance, when the robot deviates significantly from the global path, *α* can be increased to enhance path conformity; as the robot approaches the target location, *β* can be increased to strengthen target convergence; in areas with dense obstacles, *γ* can be increased to prioritize obstacle avoidance constraints; and when the terrain slope is relatively rugged, *δ* can be appropriately increased to make the evaluation function pay more attention to terrain risks, thereby ensuring driving safety.

The specific fusion strategy adopts a hierarchical architecture, where global planning and local planning work in concert: First, the improved A* algorithm is run under the global map to generate the global path *P*_*global*_; subsequently, the local target point is updated in real-time based on the robot’s position. If a dynamic obstacle occurs, making the global path infeasible, re-planning is triggered, and local terrain elevation data is obtained through the global map; under the DWA framework, candidate trajectories are generated by sampling velocity space, and a comprehensive evaluation index is calculated by combining terrain slope cost, and the optimal trajectory is selected for execution; the above process is iterated continuously until the robot reaches the target point.

However, in actual underground environments, robots often exhibit significant drift and sideslip effects during turning maneuvers. These non-ideal motion characteristics may directly affect the trajectory prediction accuracy and driving safety of DWA local planning. Therefore, future research could consider incorporating dynamic parameters such as ground adhesion coefficient and slip angle estimation into the trajectory prediction model, or enhancing trajectory prediction accuracy and control robustness in complex terrains through online state correction.

## 3. Simulation verification of path planning using improved A* algorithm

### 3.1. Performance comparison experiment of improved A* algorithm

**Fig 3:**
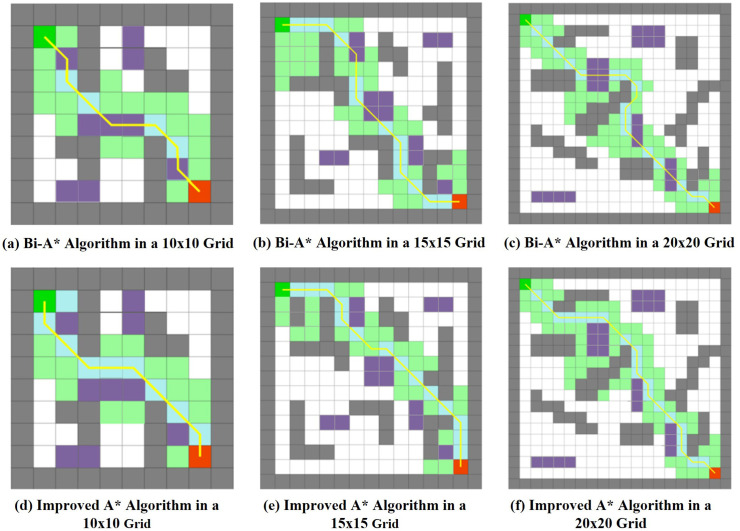
Comparison of path planning effect between the optimized algorithm and Bi-A* algorithm in this paper.

To further verify the superiority of the algorithm in this study, comparative simulation experiments were conducted on the Bi-A* algorithm and the algorithm in this study under two-dimensional grid maps of three sizes: 10 × 10, 15 × 15, and 20 × 20. The performance of the algorithms was comprehensively evaluated based on four indicators: the number of expanded nodes, path length, number of path nodes, and search time, across maps of different sizes. The planning results are shown in Fig 3.

The data comparison in Table 2 and visualization results indicate that the Bi-A* algorithm, which employs a bidirectional search strategy, can successfully plan a collision-free path. However, the generated path exhibits issues such as numerous redundant nodes, dense turning points, and weak terrain adaptability. Additionally, the total length of the path significantly increases compared to the proposed algorithm. Conversely, the algorithm in this study plans a smoother path with a significantly reduced number of turning points under the same grid scale. At the same time, it can effectively avoid high-cost grid areas, fully demonstrating superior terrain adaptability and path planning quality.

**Table 2:** Comparison of search data between two algorithms.

Map Size	Algorithm	Search Time/s	Number of expanded nodes	Path length/m	Number of waypoints
10 × 10 size	Bi-A*	1.26	33	15.6	12
	Improved A*	0.87	24	14.1	9
15 × 15 size	Bi-A*	2.31	62	21.4	28
	Improved A*	1.35	31	18.1	17
20 × 20 size	Bi-A*	5.83	122	32.7	74
	Improved A*	2.72	76	29.3	56

**Fig 4:**
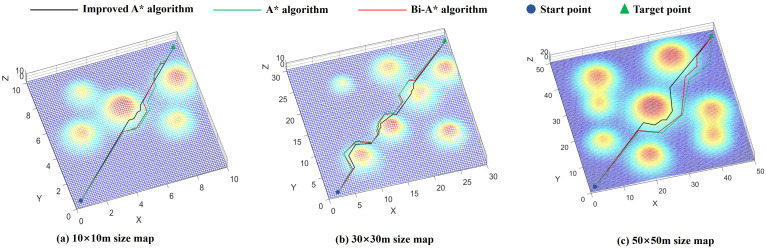
Comparison of path planning effect between the optimized algorithm and Bi-A* algorithm in this paper.

To verify the effectiveness and applicability of the algorithm in this study under sloping scenarios, a comparative experiment for 3D path planning was also designed. An unstructured 3D environment featuring slopes and undulating terrain was constructed in MATLAB 2024b, and the algorithm in this study, Bi-A* algorithm, and traditional A* algorithm was imported. The performance of each algorithm was comprehensively evaluated based on four indicators: the number of expanded nodes, path length, number of path nodes, and search time, across maps of different sizes. The planning results are shown in Fig 4.

The results indicate that the algorithm in this study, while introducing a bidirectional alternating search strategy, also incorporates a terrain risk evaluation mechanism into the cost function. This enables the robot to flexibly adjust its path based on the distribution of cost regions, effectively avoiding high-risk areas while satisfying the maximum safe slope angle constraint, thereby ensuring safe travel. In contrast, the Bi-A* algorithm, although improving search efficiency and reducing the number of expanded nodes due to the bidirectional strategy, does not consider terrain risks and misjudges low-slope areas as obstacles, leading to increased path length and planning time. The traditional A* algorithm, which employs unidirectional search, has a wider search range and covers more nodes, resulting in lower efficiency and longer time consumption in the same scenario.

As can be seen from Table 3, the algorithm in this study reduces search time by 58.4–62.3% compared to the traditional A* algorithm and by approximately 25.5–27.2% compared to the Bi-A* algorithm. In terms of the number of expanded nodes, it reduces it by 61.5–66.8% compared to the traditional A* algorithm and by approximately 35.4–37.4% compared to the Bi-A* algorithm. In summary, based on the experimental comparison data, it is evident that as the map size continues to expand, the algorithm demonstrates more significant advantages in search efficiency and path length.

**Table 3:** Comparison of search data between three algorithms.

Map Size	Algorithm	Search Time/s	Number of expanded nodes	Path length/m	Number of waypoints
10 × 10 size	A*	8.43	52	15.6	21
	Bi-A*	4.36	31	14.3	19
	Improved A*	3.17	20	12.5	17
30 × 30 size	A*	13.12	156	42.8	50
	Bi-A*	7.32	82	41.4	48
	Improved A*	5.45	51	40.1	47
50 × 50 size	A*	19.62	308	70.2	73
	Bi-A*	10.53	163	65.7	74
	Improved A*	7.81	102	60.3	71

### 3.2. Path planning experiment in simulation environment

**Fig 5:**
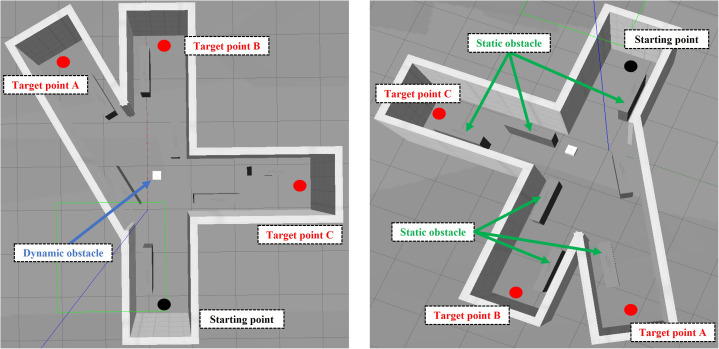
Construction of simulation platform for tracked transfer vehicle completed by gazebo software.

Taking the scene of main haulage roadway and heading face under the mine as an example, the simulated roadway environment is built in Gazebo at a ratio of 1:5, as shown in Fig 5. Among them, the size characteristics of the roadway built by the simulation platform are as follows: the width of the roadway is 1m and the total length is 6.5m. To facilitate observation, the top features are removed; There are also three representative roadways in the roadway: straight line, fork road and corner. Each roadway is 1m wide and 3m long. Target points are set at the end of each roadway, namely fork road target point a, straight line target point B and corner target point C. In addition, considering that there are all kinds of coal machinery equipment in the roadway under real working conditions, static and dynamic obstacles are arranged in the model to test the path planning ability of the robot in complex environments. Taking the long box as an example, the static obstacles are 1m × 0.25m × 1m in size, and a total of 7 groups are set in S shape; Taking the square box as an example, the dynamic obstacles are 0.25m × 0.25m × 0.25m in size and move continuously in the simulation environment.

Now combined with the improved A* algorithm and cartographer mapping algorithm proposed in this paper, the path planning experiment of tracked transfer vehicle in simulated roadway is completed. The specific workflow of the simulation is as follows: firstly, the tracked transfer vehicle model is built in the gazebo simulation environment and conFigd as a simulation platform to support path planning; Secondly, a two-dimensional map of the environment is established in the simulated roadway, the roadway entrance is set as the original starting point of the path planning, and the 2D NAV goal tool in Rviz (Robot Visualization) is used to set the target destination. The improved A* algorithm is used for global path planning to generate the optimal path from the starting point to the destination; Then, in the process of path planning, DWA algorithm, as a local path planning algorithm, adjusts the generated path in real time according to the obstacles and dynamic changes in the environment, so as to ensure that the transfer vehicle can flexibly deal with obstacles and realize safe obstacle avoidance; Finally, the transfer vehicle successfully arrived at the target point according to the optimized path.

**Fig 6:**
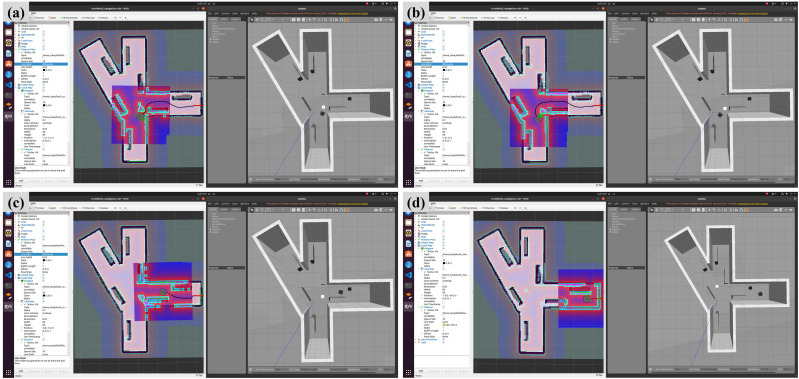
Dynamic path planning process based on BAS-A* + DWA algorithm.

**Fig 7:**
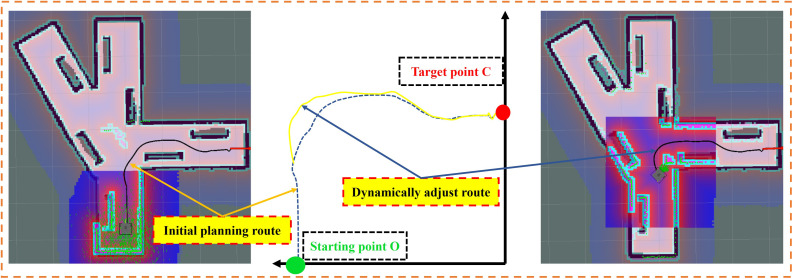
Comparison of initial planning route and dynamic adjustment route in dynamic path planning.

To effectively improve the interference with the planned expected route in the case of personnel emergence or equipment penetration, DWA algorithm can dynamically adjust the planned route in real time. Taking the simulation process as an example, the starting point of the transfer vehicle is set as the origin O, and the target end point is set as the target point C. three obstacles are set on the path respectively, and dynamic obstacles are added at the second obstacle, as shown in Fig 6. Starting from the origin, the transfer vehicle first plans a global path to the target point, then moves along the planned path and detects the information of surrounding obstacles in real time through the DWA algorithm. When it turns around two static obstacles continuously along the preset path to reach the turnout position, it detects and recognizes that the protruding obstacles appear on the initial planned route. Currently, the DWA algorithm is called to adjust the planned path in real time. The transfer vehicle monitors the position relationship between the obstacles in real time and calculates the distance Obs between the transfer vehicle and the obstacles. If Obs is greater than the collision distance doc, the corresponding speed under the simulated path will become the operation speed of the transfer vehicle at the next moment; If Obs is less than the collision distance doc, the transfer vehicle will perform a backward operation to avoid obstacles and readjust its attitude. After the dynamic obstacles pass through, it will continue to move according to the new planned path, and finally reach the target point C, as shown in Fig 7. It is proved that BAS-A* global + DWA local path planning algorithm can also meet the requirements of dynamic obstacle avoidance and new path planning under the condition of considering the penetration of obstacles.

## 4. Conclusion

To address the efficiency issue of path planning for tracked transfer robots in the complex environment of coal mines, this study proposes a path planning method suitable for underground complex tunnel environments, based on the A* algorithm and incorporating a bidirectional search strategy and a nonlinear slope penalty term. The following conclusions are drawn:

(1) Compared to the traditional A* algorithm, the algorithm in this study introduces a bidirectional alternating search strategy to enhance the search efficiency of the algorithm. Additionally, a nonlinear slope penalty term is incorporated into the cost function, enabling the robot to effectively avoid high-risk areas without exceeding the safe slope angle, thereby significantly improving the environmental adaptability of the algorithm.
(2) The results of the path planning experiment demonstrate that the algorithm proposed in this study performs well in terms of indicators such as the number of expanded nodes, path length, number of path nodes, and search time.

However, it is worth noting that in the real underground coal mine environment, perception uncertainty poses a severe challenge to the robustness of algorithms: for instance, point cloud noise and missing points caused by dust and water mist interference in lidar, as well as cumulative errors induced by slipping of odometers on rugged terrain. Specifically, perception errors may lead to distortion or delayed updating of the global map, which in turn may trigger erroneous re-planning of the global path. Meanwhile, misidentification or missed identification of local obstacles directly affects the evaluation and selection of optimal trajectories by the DWA algorithm, potentially resulting in response lag or decision bias in dynamic obstacle avoidance. Therefore, future research needs to focus on enhancing the algorithm’s resistance to perception interference: by fusing information from multiple sensors to improve the reliability of state estimation, or introducing confidence-based environmental representation and planning methods, the algorithm can quantify and handle perception uncertainty, thereby achieving safer and more stable autonomous navigation in complex underground operation scenarios. In summary, the algorithm proposed in this study provides technical support for autonomous material transportation in underground unstructured environments. Future experiments can be conducted in real mine scenarios to optimize the robustness and performance of the algorithm under extremely harsh working conditions.

## Supporting information

S1 File Data Set of this Manuscript. (7Z)
